# Responses of soil microbial biomass, microbial entropy and soil-microorganism stoichiometry imbalance to different utilization patterns in the artificial grassland of karst desertification area

**DOI:** 10.3389/fmicb.2023.1293353

**Published:** 2023-11-23

**Authors:** Yongkuan Chi, Shuzhen Song, Kangning Xiong, Gadah Albasher, Jinzhong Fang

**Affiliations:** ^1^School of Karst Science/State Engineering Technology Institute for Karst Desertification Control, Guizhou Normal University, Guiyang, China; ^2^Department of Zoology, College of Science, King Saud University, Riyadh, Saudi Arabia

**Keywords:** karst desertification, artificial grassland, soil microbial biomass, microbial entropy, stoichiometry imbalance

## Abstract

Different utilization patterns can alter the C, N, P cycles and their ecological stoichiometry characteristics in grassland soils. However, the effects of different utilization patterns on soil microbial biomass, microbial entropy and soil-microorganism stoichiometry imbalance of artificial grassland are not clear. So this study was took different utilization patterns of artificial grassland [i.e., grazing grassland (GG), mowing grassland (MG), enclosed grassland (EG)] as the research object to investigate responses of soil microbial biomass, microbial entropy and soil-microorganism stoichiometry imbalance to different utilization patterns in the karst rocky desertification control area. We found that the contents of microbial biomass carbon (MBC) and microbial biomass nitrogen (MBN) were highest in GG, and the content of microbial biomass phosphorus (MBP) was highest in EG. Soil microbial biomass entropy carbon (qMBC) and soil microbial biomass entropy nitrogen (qMBN) of GG and MG were higher than those of EG, but soil microbial biomass entropy phosphorus (qMBP) was opposite. C:N stoichiometry imbalance (C:N_imb_) was EG > GG > MG, C:P stoichiometry imbalance (C:P_imb_) was EG > MG > GG, N:P stoichiometry imbalance (N:P_imb_) was MG > EG > GG. MBN was significantly positive correlated with C:N_imb_ and C:P_imb_, MBC was significantly negative correlated with C:P_imb_, MBP was significantly negative correlated with N:P_imb_. The redundancy analysis (RDA) results showed that N:P_imb_ (*p* = 0.014), C:N_imb_ (*p* = 0.014), and C:P in the soil (C:P_soil_, *p* = 0.028) had the most significant effect on microbial entropy. EG had a significant effect on soil microbial biomass and microbial entropy. The results of this study can directly or indirectly reflect the grassland soil quality under different utilization patterns in the karst rocky desertification area, which has a certain reference value for the degraded ecosystem restoration.

## Introduction

Soil has the most diverse microbial community on the planet (Hartmann and Six, 2023), and soil microorganisms directly drive key functions in ecosystems, playing an important role in soil organic matter decomposition, nutrient cycling, C storage, maintaining soil ecosystem productivity, and determining soil fertility, crop yield, and stress tolerance (Bahram et al., 2018; Zhao et al., 2019; Sardar et al., 2021a,b). Soil microorganisms release inorganic nutrients available to plants by decomposing soil organic matter, modify nutrient availability through oxidation, reduction, dissolution and chelation processes, and store nutrients in microbial residues (Marschner and Rengel, 2007; Hu et al., 2014), ultimately driving global nutrient cycling processes (Falkowski et al., 2008). The biomass of soil microorganisms refers to the total biomass with a volume of less than 5.0 × 10^3^ μm^3^ in the soil, mainly referring to some living parts of soil organic matter such as bacteria, fungi, algae and protozoa, which is an important parameter to characterize the material cycle and energy flow in the soil ecosystem (Rogers and Tate, 2001). Meanwhile, the amount of microbial biomass can also reflect mineralization capacity of the soil and indicate the vitality of the soil (Li et al., 2018). The contents of soil organic carbon (SOC), total nitrogen (TN) and total phosphorus (TP) change little in a short time, but soil microorganisms are very sensitive to environmental changes, and their biomass stoichiometry changes with climate, biota, land use and vegetation succession. Therefore, MBC, MBN, and MBP have the characteristics of sensitivity and fast turnover rate, which are more suitable for reflecting soil fertility changes and assessing soil quality (Nair and Ngouajio, 2012; Song et al., 2022; Zhang et al., 2023). The stoichiometry characteristics of soil C, N, and P significantly influence the community structure, microbial biomass, assimilation rates and metabolic activities of soil microorganisms (Mooshammer et al., 2014; Zechmeister-Boltenstern et al., 2015; Zhou and Wang, 2016). Therefore, clarifying the stoichiometric relationship between soil and soil microorganisms is of great importance to reveal the mechanism of soil nutrient balance (Zhang et al., 2019).

Soil microbial entropy is the proportion of soil microbial biomass C, N, and P to the SOC, TN, and TP content, and is mainly used to reflect the microbial biomass that can be supported by a unit of resources (Heuck et al., 2015). Soil microbial entropy is mainly influenced by the quality and content of soil organic matter. It reflects the dynamic change of soil ecosystem by reflecting the change of soil nutrient and its use efficiency, which is a sensitive index to characterize the change of soil quality (Ji et al., 2020), and has the function of indicating the accumulation and change of soil nutrient (Rogers and Tate, 2001). The results of a study by Zhou and Wang (2016) on MBC, MBN, and MBP in 6 grassland ecosystems and 13 forest ecosystems showed that MBC, MBN, and MBP all increased significantly with the secondary succession process. The results of a study by Wu et al. (2019) on soil microbial biomass and soil microbial entropy in desert grassland showed that soil microbial biomass and soil microbial entropy will decrease with the aggravation of land degradation. Although both succession processes and the degree of degradation have different effects on soil microbial entropy, the effect of difference use patterns on soil microbial entropy in grasslands has not yet been investigated.

Soil and microbial stoichiometry imbalances, which can measure the difference between the chemical composition of microorganisms and resource, the smaller the value indicates that the higher the quality of the resource, the higher the efficiency of microbial growth, which can help to clarify the dynamic balance of nutrients between the soil and microorganisms (Mooshammer et al., 2014; Zhou and Wang, 2016). The ecological stoichiometry of soil microbial C:N:P can enhance the understanding of soil microbial ecological processes and mechanisms (Wu et al., 2019). Studies have shown that soil microbial entropy changes with the change in soil stoichiometry (Anderson and Domsch, 1990; Ding et al., 1992) and decreases with increasing the stoichiometry imbalance of soil C:N:P (Zhou and Wang, 2016). Therefore, it is of great importance to study the changes of ecological stoichiometry, microbial entropy and soil-microorganism stoichiometry imbalance under different utilization patterns to maintain ecosystem stability and sustainable development.

The karst in southwest China is an ecological security barrier area in China, with a very fragile ecological environment, and its ecological protection and economic development have received much attention from the international community (He et al., 2023). The fragile ecological environment of the region, combined with a long history of traditional agriculture such as maize and potatoes, has resulted in severe soil erosion, shallow soil layers and prominent karst desertification problems (Xiong et al., 2016). Since the end of the last century, the research results on the rocky desertification control show that returning farmland to grassland and developing herbivorous animal husbandry in the rocky desertification areas are important measures for repairing the damaged ecological environment, and are of great significance for promoting ecological reconstruction and economic development (Xiong et al., 2002). Differences in grassland use will inevitably lead to differences in nutrient ratios and elemental balances of C, N, and P in grassland soils (Song et al., 2023). At present, grazing and mowing are the main utilization patterns of karst artificial grassland, and the different utilization patterns may alter the nutrient cycling relationship between grassland soil and microorganisms formed by long-term evolution (Pei et al., 2021), thus having a profound impact on the function of grassland ecosystem. However, the ecological stoichiometry studies of soil and microorganisms in the karst area have mostly focused on forests or different land uses (Song et al., 2019; Chen et al., 2020; Lu et al., 2022; Lei et al., 2023; Wen et al., 2023), while studies on the response of soil microbial biomass, microbial entropy and soil-microorganism stoichiometry imbalance to different utilization patterns in the artificial grassland of karst area are clearly lacking. Therefore, it is urgent to clarify the characteristics of soil microbial biomass, microbial entropy and soil-microorganism stoichiometry imbalance and reveal the nutrient balance and limiting factors of soil and microbial under different grassland utilization patterns in the karst desertification area.

For this reason, we hypothesized that soil microbial biomass, microbial entropy and soil-microorganism stoichiometry imbalance would have different response characteristics to different utilization patterns in the artificial grassland of karst area. In order to solve the above conjecture, this study was took different utilization patterns of artificial grassland (i.e., GG, MG, EG) as the research object to investigate responses of soil microbial biomass, microbial entropy and soil-microorganism stoichiometry imbalance to different utilization patterns in the karst desertification control area, so as to provide theoretical support for sustainable utilization of artificial grassland in ecologically fragile areas of China and even the world.

## Materials and methods

### Study area

The study area is located in Salaxi Town, Qixingguan District, Bijie City, Guizhou Province, China (105°02′01′′–105°08′09′′E, 27°11′36′′–27°16′51′′N), which is a typical karst plateau-mountain desertification control area. The rocky desertification area is 55.931 km^2^, accounting for 64.93% of the demonstration area. The climate type belongs to the subtropical monsoon climate, with annual rainfall of about 1,000 mm, mostly concentrated in July to September. The mean temperature in July (hottest month) is 21.2°C, the mean temperature in January (coldest month) is 2.3°C, the frost-free period is about 245 days, and the average annual sunshine hours are about 1,360 h. Groundwater resources are difficult to exploit and extract, and the main sources of water for production and domestic use are springs and surface water, which is abundant in summer but severely depleted in the dry season. The vegetation is dominated by subtropical deciduous broad-leaved and evergreen broad-leaved mixed forest, accounting for about 10% of the entire area. The soil is mainly yellow soil and calcareous, formed by the weathering of carbonate rock parent material. In order to restore the ecological environment of rocky desertification caused by population pressure, we have established soil and water conservation forests, economic forests and artificial grasslands with green vegetation that has both ecological and economic benefits. The main crops are maize and potatoes, which are grown for long periods of the year, with severe soil erosion and shallow soil layers. The basic chemical properties of the soil are as follows: SOC content is ranged from 17.37 to 22.45 mg/kg, TN content is ranged from 1.74 to 2.19 mg/kg, TP content is ranged from 0.53 to 1.26 mg/kg.

### Plot setting

In April 2012, the research group established artificial grassland in the study area. The main planting method was mixed seeding, mainly with *Lolium perenne* + *Dactylis glomerata* + *Trifolium repens*, and the seed ratio was 2:2:1. After the grassland establishment, free grazing has been the main use, and the carrying capacity was 600 m^2^ per sheep unit. To reveal the difference in soil microbial biomass, microbial entropy and soil-microorganism stoichiometry imbalance under different utilization patterns, GG and MG were set up in August 2019, and EG was used as a control treatment. The area of grazing and mowing plots was about 3,000 m^2^, and the enclosure plot was about 100m^2^. Three replications were set up for three treatments, and the distance between the boundaries of each plot was more than 50 cm. The average number of grazing animals in each plot was 5 (in line with the local grazing situation), and the grazing animals were Guizhou semi-fine wool sheep about 1 year old. Except for extreme weather conditions, the grazing period was about 300 days per year. The stubble height of the mowed grassland was approximately 5 cm, and the mowing was carried out according to the normal grass phenological period or the mowing height. The EG was not used for any purpose.

### Sampling method

In mid-August 2021, 15 sampling points were set up evenly in each sample plot using the “S” shaped multi-point sampling method. After removing the litter layer from the soil surface, soil samples were taken from the surface layer (0–10 cm) using soil auger. To reduce spatial heterogeneity, soil samples from 15 sampling points were mixed into one sample, and a total of 9 soil samples were obtained. Impurities were removed from the soil samples, and the samples were divided into two parts. One part of the soil samples was naturally air dried indoors, and then passed through a 2 mm sieve for the determination of soil C, N, and P contents. The other part was placed in dry ice at −78.5°C and brought back to the laboratory for the determination of microbial C, N, and P contents.

### Samples determination

The potassium dichromate-external heating method was used to determine soil organic carbon (SOC), the sulfuric acid catalyst digestion-Kjeldahl method was used to determine total nitrogen (TN), the concentrated sulfuric acid digestion-Mo–Sb colorimetric method was used to determine total phosphorus (TP) (Bao, 2000). TN and TP were determined by the continuous flow analyzer (SYSTEA, FLOWSYS, Italy), and TP was determined by the ultraviolet spectrophotometer (Specord 200 PLUS, Analytik, Germany). The chloroform fumigation-K_2_SO_4_ leaching method was used to determine soil microbial biomass carbon (MBC), microbial biomass nitrogen (MBN), the chloroform fumigation-NaHCO_3_ extraction-Pi assay-additional Pi correction method was used to determine soil microbial biomass phosphorus (MBP) (Zhang et al., 2012; Wu et al., 2019).

### Data analysis

The ecological stoichiometric ratio of C, N, and P of soil and microorganism was expressed in mass ratios (Lin, 2010; Wu et al., 2019; Zhang et al., 2023).

Soil microbial entropy C (qMBC) = MBC/SOC × 100%.

Soil microbial entropy N (qMBN) = MBN/TN × 100%.

Soil microbial biomass entropy P (qMBP) = MBP/TP × 100%.

C:N stoichiometry imbalance (C:N_imb_) = C:N_soil_/C:N_mic_.

C:P stoichiometry imbalance (C:P_imb_) = C:P_soil_/C:P_mic_.

N:P stoichiometry imbalance (N:P_imb_) = N:P_soil_/N:P_mic_ (Wu et al., 2019; Zhang et al., 2023).

Data were analyzed using One-way ANOVA, multiple comparisons of least significant difference and Person correlation analysis in SPSS 22.0, and plotted using Origin 2018. Redundancy analysis (RDA) was performed in Canoco 5.0.

## Results

### Soil microbial biomass C, N, P and its ecological stoichiometry

As shown in Table 1, different utilization of grassland can have different effects on MBC, MBN, and MBP. The variation range of MBC content was from 270.89 to 350.24 mg/kg, and GG and MG were 22.66% and 14.17% higher than EG, respectively. The MBN content ranged from 81.53 to 142.46 mg/kg. The MBP content ranged from 7.50 to 18.82 mg/kg, and it was significantly lower in GG and MG than in EG with values of 48.24% and 137.74%, respectively. The C:N_mic_ and C:P_mic_ were highest in MG, C:N_mic_ was lowest in GG, C:P_mic_ and N:P_mic_ were lowest in EG, and C:P_mic_ was highest in GG.

**Table 1:** Soil microbial biomass C, N, P and its ecological stoichiometry of grassland under different utilization modes.

Utilization patterns	MBC (mg/kg)	MBN (mg/kg)	MBP (mg/kg)	C:N_mic_	C:P_mic_	N:P_mic_
GG	350.24 ± 37.56a	142.46 ± 10.45a	10.95 ± 37.56b	2.48 ± 0.41bc	32.34 ± 5.93b	13.11 ± 1.81a
MG	315.61 ± 31.44ab	81.53 ± 7.23c	7.50 ± 0.20c	3.88 ± 0.32a	42.09 ± 3.98a	10.86 ± 0.71a
EG	270.89 ± 11.44b	96.93 ± 10.14b	18.82 ± 0.65a	2.81 ± 0.19b	14.39 ± 0.20c	5.14 ± 0.37b

Different lowercase letters indicate significant at 0.05 probability level.

### Soil microbial entropy and stoichiometry imbalance

As shown in Table 2, anthropogenic utilization practices significantly affected qMBC and qMBN but not qMBP compared natural closure. The qMBC was significantly higher in GG and MG than in EG, but the values between GG and MG were very close. The qMBN was significantly higher in GG than that in MG and EG, and there were significant differences among the three treatments. There was no significant difference in qMBP among the three treatments. The C:N_imb_ in EG was significantly higher than that in GG and MG, and there were significant differences among the three treatments. The C:P_imb_ was significantly higher in EG than that in GG and MG. The N:P_imb_ in MG was significantly higher than that in GG, but not significantly different from that in EG.

**Table 2:** Soil microbial entropy and stoichiometry imbalance of grassland under different utilization modes.

Utilization patterns	qMBC/(%)	qMBN/(%)	qMBP/(%)	C:N_imb_	C:P_imb_	N:P_imb_
GG	0.18 ± 0.03a	0.65 ± 0.00a	0.13 ± 0.01a	3.60 ± 0.78b	0.70 ± 0.15b	0.20 ± 0.01b
MG	0.18 ± 0.01a	0.47 ± 0.01c	0.14 ± 0.01a	2.59 ± 0.20c	0.78 ± 0.05b	0.30 ± 0.01a
EG	0.12 ± 0.02b	0.53 ± 0.01b	0.15 ± 0.01a	4.38 ± 0.52a	1.24 ± 0.16 a	0.28 ± 0.01a

Different lowercase letters indicate significant at 0.05 probability level.

### Correlation analysis of soil microbial biomass and soil-microorganism stoichiometry imbalance

**Figure 1:**
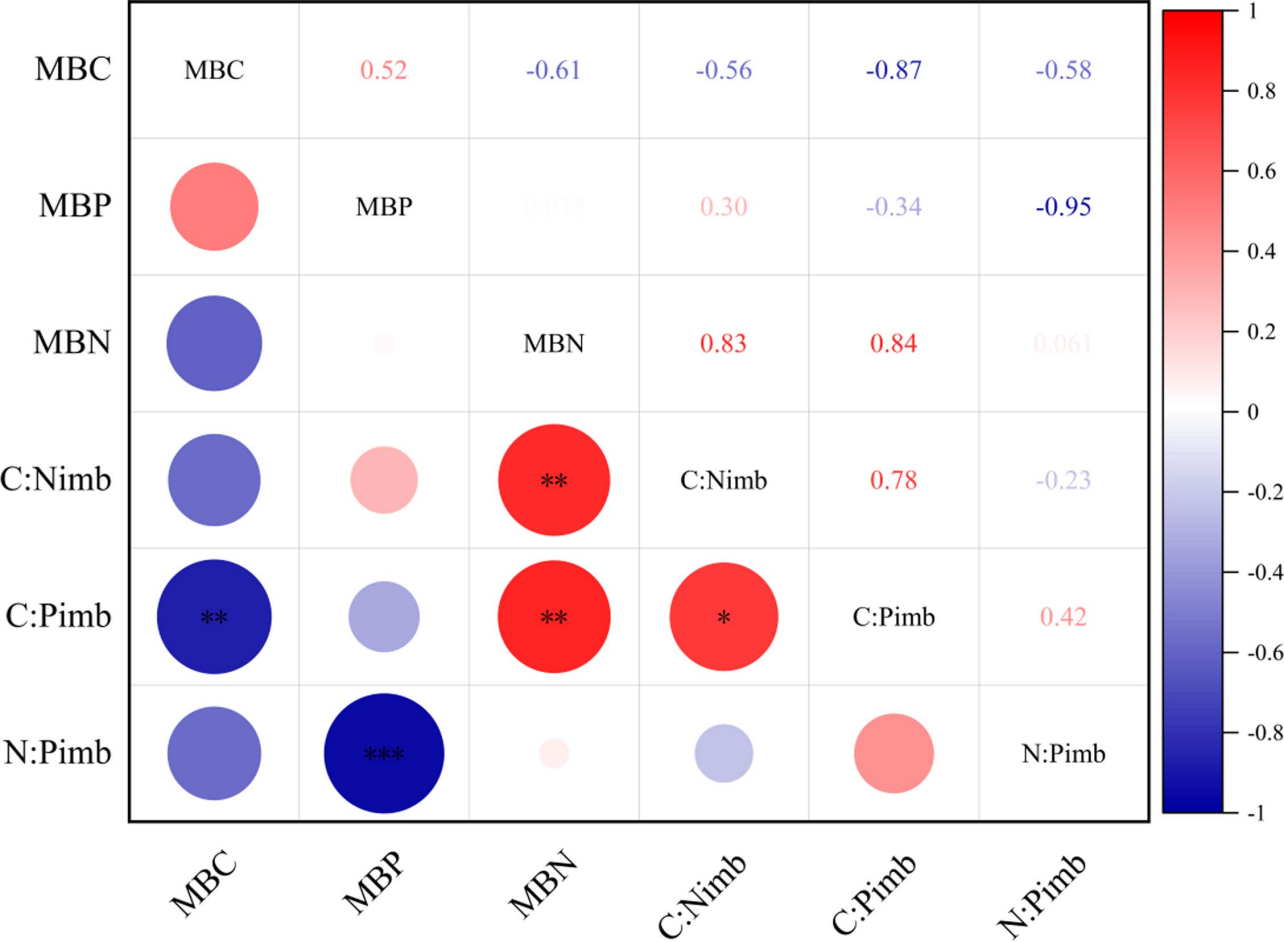
Correlation analysis between microbial biomass and soil-microorganism stoichiometric imbalance. *p <= 0.05, **p <= 0.01, ***p <= 0.001.

It can be seen from Figure 1 that there was a significant negative correlation between MBC and C:P_imb_ at the 0.01 level. MBN was positively correlated with C:N_imb_ and C:P_imb_ at 0.01 level. MBP was negatively correlated with N:P_imb_ at 0.001 level. C:N_imb_ was positively correlated with C:P_imb_ at 0.05 level.

### Relationship between soil microbial entropy and ecological stoichiometry of soil-microorganism C:N:P

**Figure 2:**
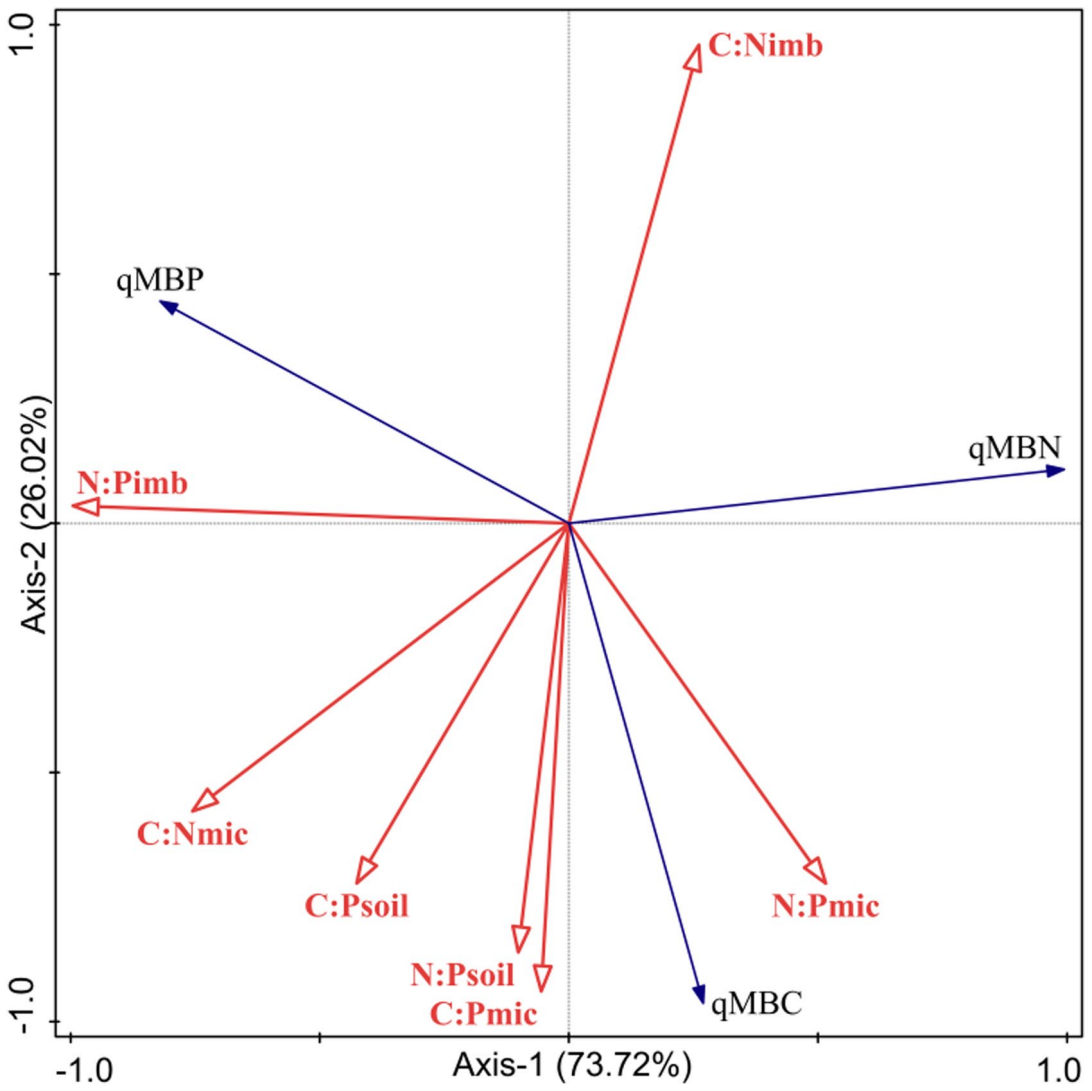
RDA analysis of soil microbial entropy and soil-microorganism C:N:P stoichiometry.

The results of the RDA analysis (Figure 2) showed that the first axis (*F* = 2.8, *p* = 0.008) and all axes (*F* = 14.29, *p* = 0.002) were statistically significantly correlated. qMBC, qMBN, and qMBP explained 73.72% and 26.02% of the variation in soil microbial entropy in the first and second axes, respectively. The results showed that the first two axes could well reflect the relationship between soil microbial entropy and soil stoichiometry imbalance, and that was mainly determined by the first axis. Among them, N:P_imb_ (*p* = 0.014), C:N_imb_ (*p* = 0.014) and C:P_soil_ (*p* = 0.028) had the most significant effect on microbial entropy. qMBC was positively correlated with C:N_mic_, C:P_soil_, N:P_soil_, C:P_mic_ and N:P_mic_, but negatively correlated with C:N_imb_ and N:P_imb_. qMBN was positively correlated with C:N_imb_ and N:P_mic_, but negatively correlated with N:P_imb_, C:N_mic_, C:P_soil_, N:P_soil_, C:P_mic_. qMBP was positively correlated with N:P_imb_, C:N_mic_, C:P_soil_, and negatively correlated with N:P_soil_, C:P_mic_, N:P_mic_.

## Discussion

### Effects of different utilization patterns on MBC, MBN, and MBP in grassland

Grassland has extremely important ecological and production functions (Bai and Cotrufo, 2022), and soil microbial biomass is an important indicator of soil development and nutrient cycling (Bastida et al., 2021). In this study, MBC, MBN, and MBP contents were different with different grassland utilization. The MBC and MBN content in GG was the highest, which may be related to that grazing livestock intensifies disturbed soil environment, promotes the accumulation of soil C and N, and improves soil organic matter and biological activity, which is consistent with the research results of Li et al. (2023). However, some studies suggest that increasing grazing intensity significantly reduces the MBN levels (Song et al., 2008). As the grazing intensity in this study was moderate, the MBN content was not affected by the grazing disturbance, but played a facilitating role, which also showed that moderate grazing will increase MBN. In this study, the MBP content in EG was significantly higher than that in GG and MG, which was closely related to the vegetation change, livestock influence, soil nutrients loss, and the litter return in the process of grassland utilization. In the process of grazing and mowing, soil nutrients, vegetation types and microbial activities will change, and the influence of artificial grazing and trampling may be the reason for the decrease in soil microbial biomass, which is consistent with the research results of Wu et al. (2019) in desert grassland of Ningxia, but inconsistent with the research of Chen (2018), who believed that enclosure measures increased P demand, and decreased soil available P content, ultimately reducing the P content of microbial biomass. The reason for the opposite results may be related to the enclosure time and environmental factors. This study was conducted in the subtropical zone of China, while Chen’s (2018) study was conducted in Inner Mongolia of northern China. The results also showed that soil microorganisms under different grassland use patterns would show different microbial activity and material metabolism ability, thus showing different MBC, MBN, MBP content, and ultimately affect the development trend of plant and soil environment.

### Effects of different utilization patterns on soil microbial entropy and soil-microorganism stoichiometry imbalance in grassland

Soil microbial entropy can reflect the microbial biomass that can be supported by a unit of soil resources, reflect differences in soil nutrients and utilization efficiency, and reveal differences in soil fertility (Anderson, 2003). In general, the soil microbial entropy is greater, soil nutrient accumulation is greater, and soil nutrient loss is greater (Sparling, 1992). In this study, the changes in qMBC, qMBN, and qMBP were different in different utilization modes. The microbial activity in the soil was greatly activated by the artificial application of GG and MG, and the qMBC was greatly increased, which was significantly higher than that of EG. Relatively, qMBN was the highest in GG and the lowest in MG, indicating that N storage increased in GG and decreased in MG. There was no significant difference in qMBP among the three treatments, but EG was the highest, indicating that its P reserve was the highest, while GG was the lowest. A previous studies found that the qMBC, qMBN, and qMBP of 84% of the ecological succession series in 19 ecological succession processes increased with the ecological succession process, indicating that the variation in soil microbial entropy can largely reflect the evolution of soil quality (Zhou and Wang, 2016). In this study, qMBN was relatively high, while qMBC and qMBP were relatively low under different grassland use patterns, indicating that C and P were relatively poor in this study area. In order to maintain C, P and other nutrients needed for plant growth, it is necessary to increase the proportion of microbial biomass in C and P to maintain a higher material metabolism capacity. With the mowing of grassland vegetation and the feeding of livestock, the grazing grassland and the mowing grassland require more C, N, P and other nutrients to accelerate the activity of SOC, which promotes the faster rate of conversion of SOC into microbial biomass, and thus the microbial entropy increases. We also found that artificial utilization measures can improve qMBC, but qMBN and qMBP need to be analyzed according to specific conditions.

Studies have shown that the higher the soil-microorganism stoichiometry imbalance value, the worse the soil quality, and the lower the growth utilization efficiency of microorganisms (Zhou and Wang, 2016). In the study, the stoichiometry imbalance of C:N_imb_, C:P_imb_ and N:P_imb_ in soil-microorganism was different under different utilization patterns. EG had the highest mean value of stoichiometric imbalance, indicating that the soil quality was poor and the growth and utilization efficiency of microorganisms was lower, while MG had the lowest mean value of stoichiometry imbalance, indicating that the soil quality was good and the growth and utilization efficiency of microorganisms was higher.

### Coupling relationship between soil microbial biomass and soil-microorganism stoichiometry imbalance

MBC, MBN, and MBP were closely related to C:P_imb_, C:P_imb_ and N:P_imb_ under different grassland use patterns. MBC and MBP were positively correlated, indicating that MBC and MBP had the same response to grassland utilization, and were strongly influenced by each other. The main reason may be that MBC and MBP are derived from the direct transformation of soil organic matter, and the transformation rate and decomposition amount of SOC and TP affect MBC and MBP. MBC and MBN were negatively correlated, which is inconsistent with the research results of northwestern Loess Plateau and Zoige sandy grassland in western Sichuan (Zhou and Wang, 2016; Zhong et al., 2017), which may be mainly related to the influence of grazing livestock, fertilization and other factors on grassland soil. The MBC was negatively correlated with C:P_imb_, that MBN was significantly positively correlated with C:N_imb_ and C:P_imb_, and the MBP was significantly negatively correlated with N:P_imb_. It can be seen that the decrease or increase of soil MBC would cause the change of MBC:MBN, MBC:MBP, MBN:MBP, and further lead to the increase of soil and microorganism stoichiometry imbalance. Our results are consistent with those of Wu et al. (2019) and Zhou and Wang (2016), that microbes can adapt to changes in soil-microorganism stoichiometry imbalance by adjusting their own biomass.

### Effect of soil-microorganism C:N:P stoichiometry on soil microbial entropy

The soil-microorganism stoichiometry imbalance can reflect the adaptation of microorganisms to soil nutrient fluctuations and the relationship between the two in the synergistic regulation of ecosystem nutrient dynamics balance (Mooshammer et al., 2014; Wu et al., 2019). In this study, the effects of the microbial ecological stoichiometry and soil-microorganism stoichiometry imbalance on soil microbial entropy were different under different utilization patterns. The qMBC was positively correlated with C:N_mic_, C:P_soil_, N:P_soil_, C:P_mic_ and N:P_mic_, and negatively correlated with C:N_imb_ and N:P_imb_, which was mainly due to the coordinated supply of soil C, N, and P nutrients required for the growth and metabolism of soil microorganisms (Zhou and Wang, 2015), which is consistent with the findings of Xu et al. (2014) and Zhou and Wang (2015). The qMBN was positively correlated with C:N_imb_ and N:P_mic_, but negatively correlated with N:P_imb_, C:N_mic_, C:P_soil_, N:P_soil_, C:P_mic_. The qMBP was positively correlated with N:P_imb_, C:N_mic_, C:P_soil_, but negatively correlated with N:P_soil_, C:P_mic_, N:P_mic_, which was consistent with the findings of Xu et al. (2014) and Zhang et al. (2023). The results showed that the balance among microbial element utilization had a certain influence on the cycling of C, N, and P in the ecosystem (Zhang et al., 2023). In this study, under relatively high soil C:N_soil_ and relatively low C:P_soil_ of EG, the microbial growth will be limited by N, resulting in the decrease of qMBC compared with GG and MG. Therefore, the decrease of microbial entropy in EG compared with GG and MG in this study may be related to the limitation of soil N, which is consistent with previous studies (Wu et al., 2019; Zhang et al., 2023). In general, the variation characteristics of soil microbial biomass, microbial entropy and stoichiometry imbalance can directly or indirectly reflect the soil quality of grassland under different utilization patterns, and provide scientific reference for sustainable management of degraded ecosystem grassland in the karst desertification area.

## Conclusion

This study was investigated soil microbial biomass, microbial entropy and soil-microorganism stoichiometry imbalance of artificial grassland under different utilization patterns in the karst control area. We came to the following conclusion: Soil microbes showed different microbial activity and ability to metabolize materials under different grassland use patterns with different MBC, MBN, MBP content. The content of MBC and MBN was highest in GG, and the MBP content was highest in EG. GG and MG treatments strongly activated soil microbial activity, and significantly increased qMBC, which was significantly higher than that in EG, indicating that artificial utilization measures can improve qMBC. The mean value of soil-microorganism stoichiometry imbalance in EG was the highest, while that in MG was the lowest. The decrease or increase of MBC content would lead to the change of MBC:MBN, MBC:MBP, MBN:MBP, and finally lead to the increase of soil and microbial stoichiometry imbalance. N:P_imb_ (*p* = 0.014), C:N_imb_ (*p* = 0.014) and C:P_soil_ (*p* = 0.028) had the most significant effect on microbial entropy. The results of this study may directly or indirectly reflect the status of grassland soil quality under different utilization patterns in the karst desertification area, and have some reference value for the study of degraded ecosystem restoration.

## Data availability statement

The datasets presented in this study can be found in online repositories. The names of the repository/repositories and accession number(s) can be found at: https://doi.org/10.6084/m9.figshare.24129651.

## Author contributions

YC: Conceptualization, Data curation, Funding acquisition, Investigation, Resources, Visualization, Writing – original draft, Writing – review & editing, Formal analysis, Methodology, Project administration, Software, Supervision, Validation. SS: Conceptualization, Data curation, Validation, Visualization, Writing – original draft, Formal analysis, Funding acquisition, Project administration, Resources. KX: Conceptualization, Formal analysis, Funding acquisition, Investigation, Supervision, Writing – original draft. GA: Data curation, Formal analysis, Funding acquisition, Visualization, Writing – original draft, Writing – review & editing. JF: Supervision, Validation.
